# Prospective Analysis of the Feasibility of the PASCAL System for Transcatheter Mitral Valve Repair (OneForAll‐Registry)

**DOI:** 10.1002/ccd.31468

**Published:** 2025-03-12

**Authors:** Katharina Hellhammer, Florian Schindhelm, Matthias Riebisch, Rolf Alexander Janosi, Alexander Y. Lind, Matthias Totzeck, Peter Luedike, Tienush Rassaf, Amir Abbas Mahabadi

**Affiliations:** ^1^ West German Heart and Vascular Center, Department of Cardiology and Vascular Medicine University Hospital Essen Essen Germany

## Abstract

**Background:**

Mitral valve transcatheter edge‐to‐edge repair (M‐TEER) is increasingly applied in patients with high surgical risk. We aimed to evaluate whether the PASCAL system can be applied in an all‐comers cohort irrespective of the underlying anatomy and whether technical features influence therapeutic success.

**Methods:**

In this prospective, observational study we enrolled consecutive patients (*n* = 80) with mitral regurgitation (MR) 3+ and 4+ scheduled for M‐TEER. All patients were allocated to be treated with the PASCAL system irrespective of the underlying anatomy. Complexity of mitral valve anatomy was assessed according to the proposed complexity scale and the ESC/EACTS complexity scale. All patients underwent intraprocedural analysis of application of technical features of the PASCAL technology and 1‐year follow‐up.

**Results:**

M‐TEER was successful in 98.8% of the patients. Reduction of MR 3+/4+ to MR≤ 2+ was achieved in 92.5%. Independent leaflet grasping was applied in 60.0% of procedures. The median number of grasping attempts was 4.0 ± 3.1 for the first device. Classification in degenerative, functional, or mixed MR did not correlate with procedure time and grasping attempts. In contrast, the presence of complexity criteria was linked with a longer procedure time (*p* = 0.002) and required more grasping attempts (*p* = 0.010).

**Conclusions:**

M‐TEER with the PASCAL technology was possible in 98.8% of consecutive, all‐comers patients irrespective of the underlying anatomy. Technical features were applied frequently with increasing application in complex anatomical cases. Classifications taking the anatomical complexity into account rather than the pathophysiological entity of MR seem superior to predict the technical challenges of a M‐TEER procedure.

**Abbreviations**
CABG: coronary artery bypass grafting
CAD: coronary artery disease
COPD: chronic obstructive pulmonary disease
DMR: degenerative mitral regurgitation
FMR: functional mitral regurgitation
MMR: mixed mitral regurgitation
M‐TEER: mitral valve transcatheter edge‐to‐edge repair

## 1 Introduction

Mitral valve transcatheter edge‐to‐edge repair (M‐TEER) has become an effective therapeutic option for patients presenting with symptomatic severe mitral regurgitation (MR) considered to be at high risk for surgical interventions. Besides symptomatic improvement, patients' prognosis can be improved resulting in less hospitalization for heart failure and prolonged survival [1, 2]. Current devices available for M‐TEER are the MitraClip system (Abbott Vascular) and the PASCAL transcatheter mitral valve repair system (Edwards Lifesciences). The PASCAL device allows independent leaflet grasping and has a nitinol spacer between the paddles to reduce tension on the leaflets. It has been claimed that the PASCAL device provides a more user‐friendly steering mechanism [3]. However, varying anatomical characteristics such as flail gaps, tethering and calcification at the leaflet tips may complicate the grasping of the leaflets and the positioning of the device. For patients with degenerative MR (DMR) and presence of complex mitral valve anatomy, the prospective CLASP IID registry showed that the PASCAL system enabled high procedural success rates and long‐term MR reduction [4]. Whether these results can be transferred into an all‐comers real world cohort and whether technical features influence therapeutic success has not been confirmed so far. We therefore aimed to evaluate the feasibility of the PASCAL technology for transcatheter mitral valve repair in an all‐comers cohort.

## 2 Methods

### 2.1 Study Cohort

In a single‐center prospective observational study we analyzed 80 consecutive patients with MR 3+ and 4+ scheduled for M‐TEER after interdisciplinary heart team consensus at the West German Heart and Vascular Center after October 2020 till September 2021. All patients were allocated to be treated with the PASCAL System (PASCAL and PASCAL Ace) irrespective of the underlying anatomy. The procedures were performed by two interventional cardiologists with both experience > 3 years in M‐TEER. All patients underwent intraprocedural analysis of application of technical features of the PASCAL technology (independent grasping, leaflet optimization) and complications like leaflet injury, single leaflet device attachment or chordal entrapment were assessed. The study was approved by the local ethics committee of the University Hospital Essen‐Duisburg, is listed at Clinical Trials (NCT04473092) and was performed in accordance with the Declaration of Helsinki. All patients provided written informed consent for data acquisition and analysis.

### 2.2 Baseline and Procedural Characteristics

Baseline characteristics and pre‐procedural echocardiographic data were analyzed. MR etiology was classified as atrial functional MR, ventricular functional MR, DMR or mixed MR (MMR). Further, anatomical complexity criteria according to Hausleiter et al. [5] as well as the ESC/EACTS MR complexity scale [6, 7] were evaluated. The number of grasping attempts, measures of leaflet optimization, procedural duration, and post‐procedural outcomes were assessed in a standardized manner. All patients underwent transthoracic echocardiography before discharge. A follow‐up transthoracic echocardiography 1 year after the procedure was conducted.

### 2.3 Statistical Analysis

Continuous variables are expressed as mean ± standard deviation and compared using analysis of variance and Kruskal–Wallis test depending on variable distribution. Categorical variables were compared using *χ* ^2^ testing or Fisher's exact test. All statistical analyses were performed using SPSS Statistic (IBM Inc.). A *p* < 0.05 was considered statistically significant.

## 3 Results

From 10/2020 to 09/2021, 80 patients (mean age: 74 ± 6.7 years, 59% male) were included in the study. M‐TEER was successfully performed in 98.8% of the patients. In one patient, M‐TEER was aborted due to an increased intraprocedural gradient of 8 mmHg. 75% of the patients were implanted with the PASCAL implant, 17.5% of the patients were implanted with PASCAL Ace, and 7.5% of the patients were implanted with both devices. Underlying mitral valve pathology was ventricular functional MR in 36.3% of the patients whereas 30.0% of the patients presented with DMR, 8.8% of the patients with atrial functional MR and MMR was found in 25.0% of the patients. Baseline characteristics are shown in Table 1. At least one complexity criteria was met in 61.3% of the patients. The most common anatomical complexity was the presence of ≥ 2 independent jets in 41.3% of the patients. In 26.3% of the patients, a significant jet in the commissural area was found and severe calcification in the grasping area was present in 21.3% of the patients. Prevalence of anatomical complexity criteria is shown in the Graphical Abstract (Central Illustration Illustration 1, 1).

**Table 1:** Baseline characteristics.

	All patients (*n* = 80)
Male, *n* (%)	47 (59.0)
Age, years	74 ± 3.2
STS score (%)	5.5 ± 5.4
CAD, *n* (%)	54 (67.6)
Previous CABG, *n* (%)	13 (16.5)
Atrial fibrillation, *n* (%)	51 (63.7)
COPD, *n* (%)	9 (11.3)
CRT, *n* (%)	10 (12.5)
Previous valve intervention, *n* (%)	4 (5.0)
Presence of heart failure, *n* (%) − HFrEF, *n* (%) − HFmrEF, *n* (%) − HFpEF, *n* (%)	70 (87.5)
	30 (37.5)
	20 (25.0)
	20 (25.0)
Chronic kidney disease, *n* (%)	20 (25.0)
Atrial FMR, *n* (%)	7 (8.8)
Ventricular FMR, *n* (%)	29 (36.3)
DMR, *n* (%)	24 (30.0)
MMR, *n* (%)	20 (25.0)

Abbreviations: CABG = coronary artery bypass grafting, CAD = coronary artery disease, COPD = chronic obstructive pulmonary disease, CRT = cardiac resynchronization therapy, DMR = degenerative functional mitral regurgitation, FMR = functional mitral regurgitation; HFmrEF = heart failure with midly reduced ejection fraction, HFpEF = heart failure with preserved ejection fraction, HFrEF = heart failure with reduced ejection fraction, MMR = mixed mitral regurgitation.

**Central Illustration 1:**
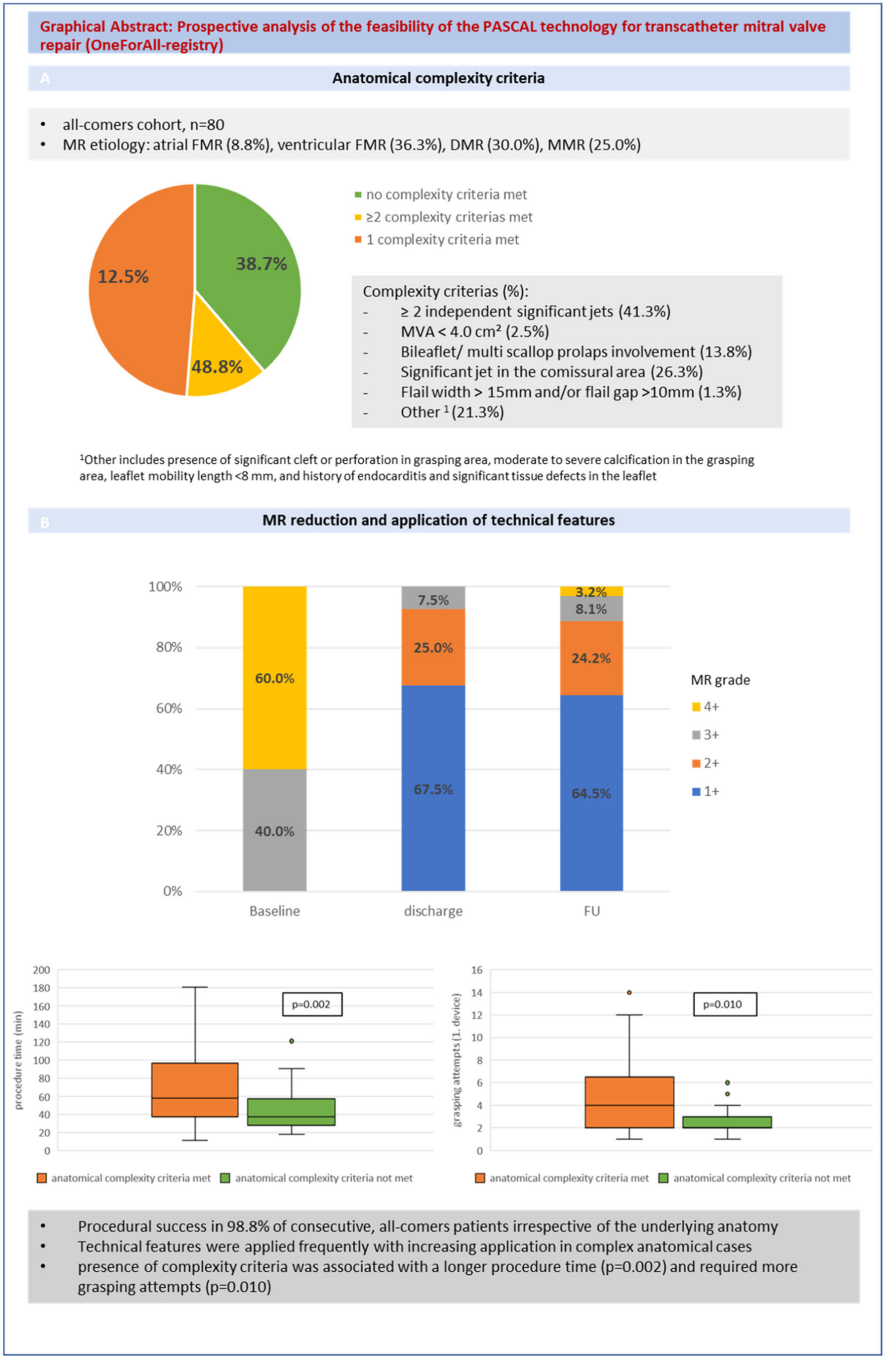
Prospective analysis of the feasibility of the PASCAL technology for transcatheter mitral valve repair (OneForAll‐registry). [Color figure can be viewed at wileyonlinelibrary.com]

Reduction of MR 3+/4+ to MR ≤ 2+ was achieved in 92.5% at discharge and 88.7% after 1 year (Central Illustration Illustration 1, 1). Independent leaflet grasping was applied in 60.0% of cases. The median number of grasping attempts was 4.0 ± 3.1 for the first device, and 2.8 ± 1.9 for the second device. Leaflet optimization was performed in 60.0% of cases for the first device, and 13.8% of the cases for the second device. Procedural data are shown in Table 2. Technical features such as independent grasping and leaflet optimization were more frequently applied in patients where at least one complexity criteria was fulfilled (Figure Illustration 1, 1). No cases with leaflet injury, single leaflet detachment or chordal entrapment were documented. Classification in DMR, functional MR or MMR did neither correlate with complexity of procedure as illustrated by grasping attempts (*p* = 0.212) nor procedure length (*p* = 0.287). In contrast, classification according to the proposed Hausleiter‐complexity scale and the ESC/EACTS complexity scale were statistically associated with more grasping attempts (*p* = 0.010; *p* = 0.014, for Hausleiter‐complexity and ESC/EACTS complexity, respectively) and longer procedure time (*p* = 0.002; *p* = 0.002, for Hausleiter‐complexity and ESC/EACTS complexity, respectively) as shown in Figure 2 and Figure 3.

**Table 2:** Procedural data.

Procedure time, min	60.0 ± 9.3
Fluoroscopy time, min	16.2 ± 9.3
Independent grasping applied, *n* (%)	48 (60.0%)
Number of grasping attempts (1. device)	4.0 ± 3.1
Number of grasping attempts (2. device)	2.8 ± 1.9
Leaflet optimization applied (1. device), *n* (%)	48 (60%)
Leaflet optimization applied (2. device), *n* (%)	11 (13.8%)

**Figure 1:**
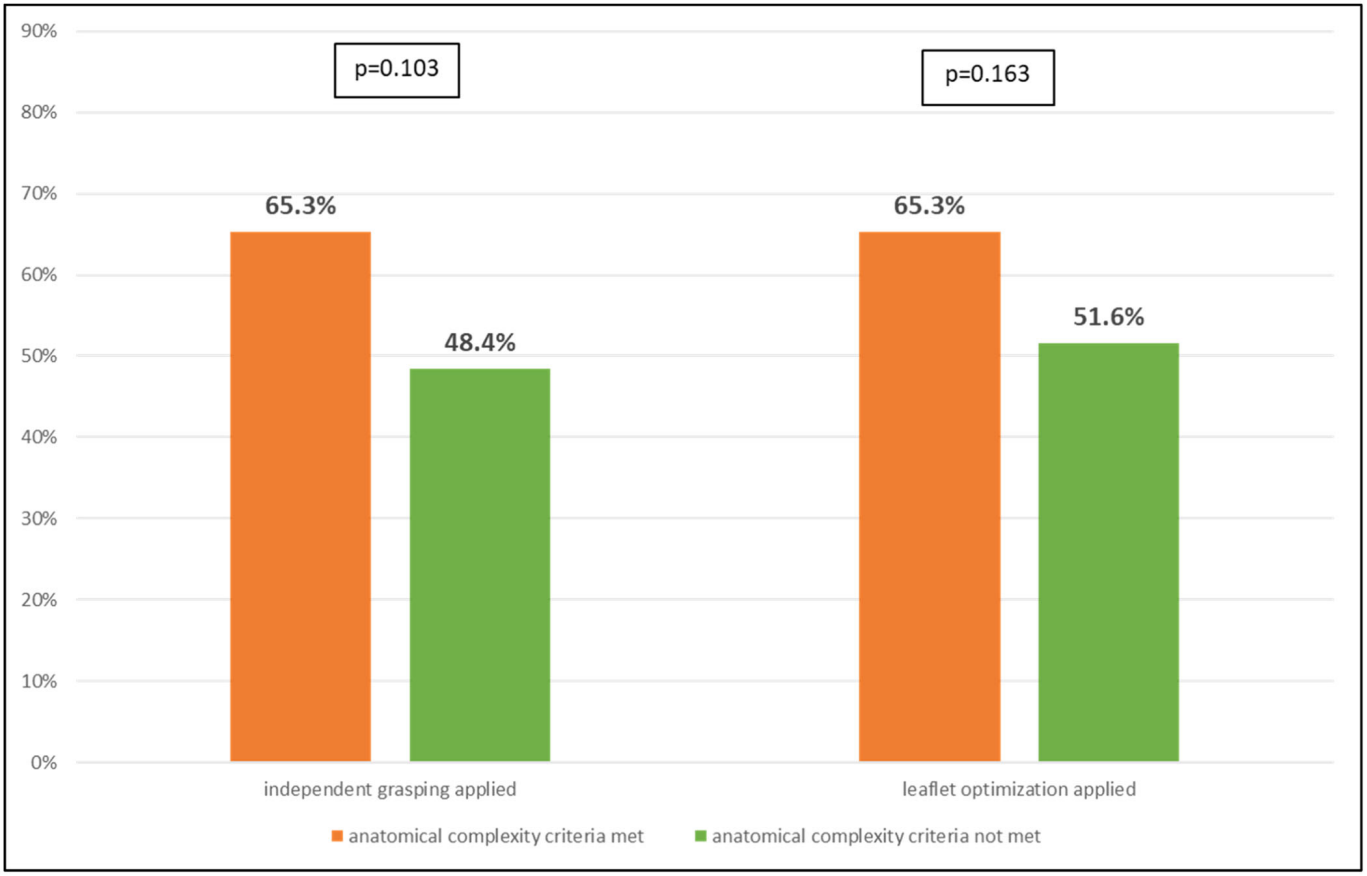
Application of technical features according to the presence of anatomical complexity criteria. [Color figure can be viewed at wileyonlinelibrary.com]

**Figure 2:**
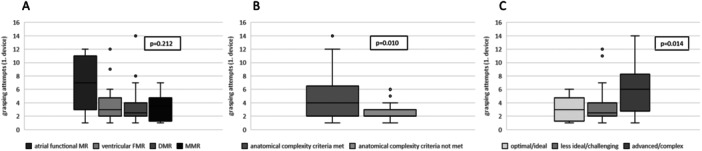
Grasping attempts according to MR etiology (A), complexity of mitral valve anatomy according to Hausleiter et al. (B) and ESC/EACTS complexity scale (C). MR = mitral regurgitation, DMR = degenerative mitral regurgitation, MMR = mixed mitral regurgitation.

**Figure 3:**
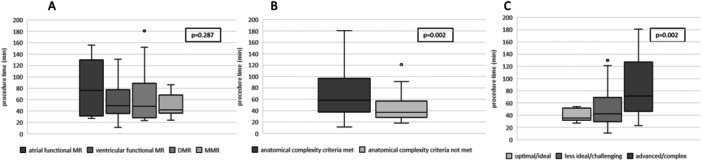
Procedure time according to MR etiology (A), complexity of mitral valve anatomy according to Hausleiter et al. (B) and ESC/EACTS complexity scale (C). MR = mitral regurgitation, DMR = degenerative mitral regurgitation, MMR = mixed mitral regurgitation.

## 4 Discussion

The current study was conducted to evaluate whether the PASCAL system can be applied for transcatheter mitral valve repair irrespective of the underlying anatomy of the mitral valve. From our data, we conclude as follows: (1) the PASCAL technology is safe with no cases with leaflet injury, single leaflet detachment, or chordal entrapment documented, (2) mitral valve repair using the PASCAL system is highly feasible in the vast majority of cases with a successful procedure in 98.8% of the patients, irrespective of the underlying anatomy of the mitral valve and stable results even after 1 year of follow‐up, (3) technical features like independent grasping or leaflet optimization were applied frequently with increasing application in complex anatomical cases, (4) identifying anatomical aspects featuring complex mitral valve anatomy may help to predict the technical challenges of a M‐TEER procedure rather than the pathophysiological entity of MR.

Transcatheter mitral valve edge‐to‐edge repair can be challenging as mitral valve anatomy and image quality may aggravate the intervention. MR is a heterogeneous condition with several underlying etiologies and many anatomical variations. Two devices, the MitraClip system and the PASCAL system, are currently approved for M‐TEER. The more recently introduced PASCAL device features a nitinol spacer between the paddles easing the strain on the leaflets [8]. Further, independent grasping of the leaflets can be applied. Since the EVEREST II Trial in 2012 [9] has proposed favorable anatomical characteristics for M‐TEER, earlier studies have shown the safety and applicability of the device as well in complex anatomies [5, 10, 11, 12]. Whether the classification of MR and evaluation of anatomical aspects correlates with procedural maneuvers has not been analyzed so far. Current classification of MR according to the ESC/EACTS Guidelines [7] differentiates between primary or DMR and secondary or functional MR, even though a mixed etiology can be observed. Atrial functional MR describes a newly established disease entity in which MR occurs secondary to left atrial disease without left ventricular dilatation. In our study population, DMR was found in 30% of the patients whereas 45% presented with atrial or ventricular FMR and 25% with MMR which is in line with previous data [11]. We did not observe an association between classification of MR and procedural aspects as procedure time or grasping attempts. Therefore, complexity of M‐TEER interventions is not based on MR classification into FMR, DMR or MMR when using the PASCAL system.

In addition to the classification of MR, anatomical complexity seems to indicate procedural challenges as technical features are more frequently applied. This may be taken into consideration when planning the procedure with regard to operator experience. When allocating patient's mitral valve anatomy according to ESC/EACTS Guidelines to “optimal/ideal,” “less ideal/challenging,” and “advanced/complex” we found prolonged procedure time and more grasping attempts in complex cases. Characterizing patients before the procedure in a more “anatomical” way on top to the pathophysiological entity may help to identify challenging cases. However, independent of longer procedural time and more grasping attempts, complexity criteria were still associated with good results with a high procedural success using the PASCAL system. Our data suggests that the PASCAL system with its flexibility due to the nitinol frame and the option for independent grasping, allows for complex anatomical features to be treated successfully.

The study has several limitations. First, it is a single center study and results should be confirmed in additional multi‐center trials with a larger patient cohort. Second, though anatomical complexity criteria may have the most relevant impact on procedural success, other factors like image quality or procedural aspects such as anesthesiologic considerations may as well influence the procedure and periprocedural maneuvers. Third, given the relatively small sample size, specific anatomical settings such as Barlow's disease or Fabry disease were not represented in a sufficient number of cases to allow for reliable conclusions in these cohorts.

In conclusion, M‐TEER with the PASCAL system was successful in 98.8% of consecutive, all‐comer patients irrespective of the underlying anatomical complexity and MR etiology. MR reduction was achieved in the vast majority of patients with trace or mild MR at discharge and stable results after 1 year. Technical features like independent grasping or leaflet optimization were applied frequently with increasing application in complex anatomical cases, but there was no difference in the use of these features by MR etiology. The results of the study indicate that identification of complex anatomy is more important than MR etiology when predicting technically challenging M‐TEER procedures.

## Disclosure

P.L. received honoraria for consulting and lectures from Edwards Lifesciences Irvine, US‐CA and refund of traveling expenses from Abbott Illinois, US‐IL. A.Y.L. received honoraria for lectures from Edwards Lifesciences Irvine, US‐CA outside the submitted study. A.A.M. received honoraria for consulting and lectures from Edwards Lifesciences Irvine. Tienush Rassaf has received honoraria, lecture fees, and grant support from Edwards Lifesciences, AstraZeneca, Bayer, Novartis, Berlin Chemie, Daiicho‐Sankyo, Boehringer Ingelheim, Novo Nordisk, Cardiac Di‐mensions, and Pfizer, all unrelated to this work. He is cofounder of Bimyo GmbH, a company that develops cardioprotective peptides.

## Conflicts of Interest

The authors declare no conflicts of interest.

## Data Availability

The data that support the findings of this study are available on request from the corresponding author. The data are not publicly available due to privacy or ethical restrictions.
